# Comorbidity health pathways in heart failure patients: A sequences-of-regressions analysis using cross-sectional data from 10,575 patients in the Swedish Heart Failure Registry

**DOI:** 10.1371/journal.pmed.1002540

**Published:** 2018-03-27

**Authors:** Claire A. Lawson, Ivonne Solis-Trapala, Ulf Dahlstrom, Mamas Mamas, Tiny Jaarsma, Umesh T. Kadam, Anna Stromberg

**Affiliations:** 1 Diabetes Research Centre, Leicester University, Leicester, United Kingdom; 2 Institute for Applied Clinical Sciences, Keele University, Keele, United Kingdom; 3 Department of Medical and Health Sciences, Linköping University, Linköping, Sweden; 4 Department of Cardiology, Linköping University, Linköping, Sweden; 5 Keele Cardiovascular Research Group, Centre for Prognosis Research, Institute of Primary Care and Health Sciences, Keele University, Keele, United Kingdom; 6 Department of Social and Welfare Studies, Linköping University, Linköping, Sweden; 7 Mary MacKillop Institute for Health Research, Australian Catholic University, Melbourne, Victoria, Australia; National University of Singapore, SINGAPORE

## Abstract

**Background:**

Optimally treated heart failure (HF) patients often have persisting symptoms and poor health-related quality of life. Comorbidities are common, but little is known about their impact on these factors, and guideline-driven HF care remains focused on cardiovascular status. The following hypotheses were tested: (i) comorbidities are associated with more severe symptoms and functional limitations and subsequently worse patient-rated health in HF, and (ii) these patterns of association differ among selected comorbidities.

**Methods and findings:**

The Swedish Heart Failure Registry (SHFR) is a national population-based register of HF patients admitted to >85% of hospitals in Sweden or attending outpatient clinics. This study included 10,575 HF patients with patient-rated health recorded during first registration in the SHFR (1 February 2008 to 1 November 2013). An a priori health model and sequences-of-regressions analysis were used to test associations among comorbidities and patient-reported symptoms, functional limitations, and patient-rated health. Patient-rated health measures included the EuroQol–5 dimension (EQ-5D) questionnaire and the EuroQol visual analogue scale (EQ-VAS). EQ-VAS score ranges from 0 (worst health) to 100 (best health). Patient-rated health declined progressively from patients with no comorbidities (mean EQ-VAS score, 66) to patients with cardiovascular comorbidities (mean EQ-VAS score, 62) to patients with non-cardiovascular comorbidities (mean EQ-VAS score, 59). The relationships among cardiovascular comorbidities and patient-rated health were explained by their associations with anxiety or depression (atrial fibrillation, odds ratio [OR] 1.16, 95% CI 1.06 to 1.27; ischemic heart disease [IHD], OR 1.20, 95% CI 1.09 to 1.32) and with pain (IHD, OR 1.25, 95% CI 1.14 to 1.38). Associations of non-cardiovascular comorbidities with patient-rated health were explained by their associations with shortness of breath (diabetes, OR 1.17, 95% CI 1.03 to 1.32; chronic kidney disease [CKD, OR 1.23, 95% CI 1.10 to 1.38; chronic obstructive pulmonary disease [COPD], OR 95% CI 1.84, 1.62 to 2.10) and with fatigue (diabetes, OR 1.27, 95% CI 1.13 to 1.42; CKD, OR 1.24, 95% CI 1.12 to 1.38; COPD, OR 1.69, 95% CI 1.50 to 1.91). There were direct associations between all symptoms and patient-rated health, and indirect associations via functional limitations. Anxiety or depression had the strongest association with functional limitations (OR 10.03, 95% CI 5.16 to 19.50) and patient-rated health (mean difference in EQ-VAS score, −18.68, 95% CI −23.22 to −14.14). HF optimizing therapies did not influence these associations. Key limitations of the study include the cross-sectional design and unclear generalisability to other populations. Further prospective HF studies are required to test the consistency of the relationships and their implications for health.

**Conclusions:**

Identification of distinct comorbidity health pathways in HF could provide the evidence for individualised person-centred care that targets specific comorbidities and associated symptoms.

## Abstract 2

Using cross-sectional data from the Swedish Heart Failure Registry, Claire Lawson and colleagues examine the comorbidity patient-rated health pathways in heart failure patients

## Author summary

**Why was this study done?:**

- Heart failure is an increasingly common condition, and patients often experience persistent symptoms and poor quality of life, even when they are receiving the best possible treatment for their heart failure.
- Most heart failure patients have other conditions that dominate their health experience, yet heart failure treatment focuses on their cardiovascular status.
- There is a lack of understanding about the relationships among different comorbidities and quality of life in heart failure, which are important to guide individualised treatment plans for patients.

**What did the researchers do and find?:**

- We used an established health-related quality of life model to develop and test a new heart failure health model that included the most common heart failure comorbidities.
- We tested this model by examining the postulated relationships among comorbidities, symptoms and functional limitations reported by patients, and their overall health experience, using a national register of heart failure patients in Sweden.
- We found that non-cardiovascular comorbidities were associated with much higher overall symptom burden and more severe symptoms than cardiovascular comorbidities.
- Predominant symptoms for cardiovascular comorbidities were pain and anxiety, whereas for non-cardiovascular comorbidities they were shortness of breath and fatigue.
- Heart failure optimising therapies did not influence these symptoms, functional limitations, or quality of life.

**What do these findings mean?:**

- Current guidelines in heart failure focus on improving cardiovascular status in response to common heart failure symptoms (shortness of breath, fatigue, and leg swelling).
- Our study shows that for some patients, these symptoms might be driven by non-cardiovascular conditions such as diabetes and renal disease, rather than their cardiovascular status. We found that cardiovascular comorbidities were more likely to be associated with pain and anxiety than shortness of breath or fatigue.
- To improve health-related quality of life, heart failure guideline-driven care needs to include optimal management of the most prevalent non-cardiovascular comorbidities and routine management of pain and anxiety or depression.
- To provide individualised patient care, guidelines need to better align symptoms with the cardiovascular and non-cardiovascular status of the patient.

## Introduction

Heart failure (HF) is a complex clinical syndrome of multiple symptoms, functional impairments, and poor health-related quality of life (HRQoL). With modern therapies, HF patients are now living longer but with a potentially higher symptom burden [1] that can be worse compared to people with other chronic diseases including cancer [2]. Inadequate symptom control and poor HRQoL are significant drivers of hospitalisations, readmissions, and death in HF [3,4].

HF patients are usually older, with a high number of comorbidities, and a third of patients report that other medical conditions dominate their health experience [5], yet guideline-driven symptom management in HF focuses on cardiovascular status [6]. Persisting symptoms and poor HRQoL after optimisation of HF treatments [7] suggest that comorbidities may be an important determinant of health and that non-cardiovascular comorbidities may be associated with HF-related symptoms [8]. However, the evidence is inconsistent, with some HF studies showing associations between comorbidities and HRQoL [9–11] and others showing no such associations [12,13]. Whilst comorbidities are consistent predictors of morbidity and mortality in HF [14], their interrelationships with symptoms, functional limitations, and overall health have not been explored. We used the Swedish Heart Failure Registry (SHFR), which is one of the largest population-based HF registers, to routinely collect patient-reported outcomes [15] to investigate the interplay among HF comorbidities, symptoms, functional limitations, and patient-rated health.

## Methods

### Study population

The SHFR is a national population-based register of all HF patients admitted to hospitals or attending outpatient clinics in participating primary care units [15]. The register collects HRQoL data measured using the EuroQol–5 dimension (EQ-5D) questionnaire, including the EuroQol visual analogue scale (EQ-VAS), at baseline registration, as well as patient demographics and clinical and healthcare information. We obtained first entry data for all HF patients included in the SHFR from 1 February 2008 to 1 November 2013 with EQ-5D recorded at baseline.

Establishment of the registry and analysis of data were approved by a multisite ethics committee and conform to the Declaration of Helsinki. Individual patient consent is not required, but patients are informed of entry into national registries and can opt out. Access to the SHFR was granted following project approval (S1 Proposal) by the SwedeHF Research Board. The original statistical analysis plan was based on standard linear and logistic regression analyses. The sequences-of-regressions approach was adopted at the early stages of data acquisition to provide a novel method of analysis to deal effectively with the complexity underlying the analysis of postulated relationships among many variables. This study is reported as per the RECORD guidelines (S1 Checklist).

### Model of HF pathways to patient-reported outcomes

**Fig 1:**
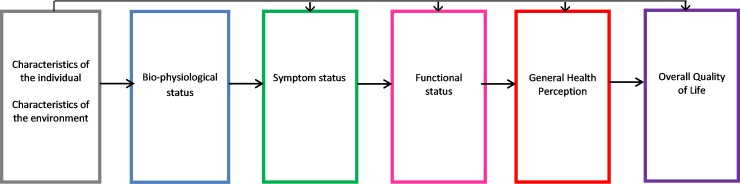
Wilson and Cleary’s health-related quality of life conceptual model. Wilson and Cleary’s model for health-related quality of life [16], revised with permission from *JAMA* [17]. Wilson and Cleary’s general conceptual model of health links objective biological measurements to the subjective consequences of disease as perceived by patients. In this model, Wilson and Cleary propose causal linkages between 5 health domains: bio-physiological status, symptoms, functional status, general health perception, and quality of life. The arrows represent dominant causal relationships. Reciprocal relationships between the variables are recognised to exist but are not represented, and we did not consider these. The revised version extends the influence of individual and environmental characteristics to all health domains in the model.

**Fig 2:**
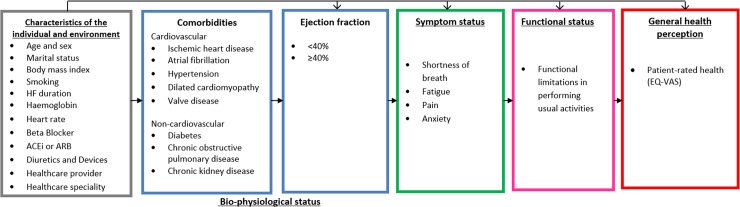
Hypothetical HF health model. Model based on a revised version of Wilson and Cleary’s health-related quality of life conceptual model [16,17]. The arrows represent direct relationships for patient and environmental factors as well as 4 of the 5 health domains: bio-physiological status (comorbidities), symptoms, functional status, and general health perception. Only arrows between adjacent domains are displayed, but it is postulated that each domain may have other direct relationships with any of the proceeding domains, and patient and environmental factors are related to every domain. ACEi, angiotensin converting enzyme inhibitor; ARB, angiotensin II receptor blocker; EQ-VAS, EuroQol–5 dimension visual analogue scale; HF, heart failure.

Previously, an a priori model by Wilson and Cleary [16] hypothesized a potential pathway from chronic disease to patient-reported outcomes and patient-rated health. The model proposes a pathway of linkages between 5 domains: (i) bio-physiological status, (ii) symptoms, (iii) functional status, (iv) general health perception (i.e., patient-rated health), and (v) quality of life (Fig 1). We used this concept to develop a HF health model with a focus on comorbidities to test the hypothesis that the potential mechanisms underlying health in HF differ among selected comorbidities. We included cardiovascular conditions related to the aetiology of HF (defined as cardiovascular comorbidities) to compare with other, non-cardiovascular comorbidities. Wilson and Cleary’s model has since been adapted to take account of patient and environmental factors that might influence any stage of the model, including bio-physiological status [17]. We used this adapted version, given the clear link between these factors and the development of both HF and comorbidities in older age. All variables selected to include in the model were chosen by an expert group including HF specialists (Fig 2). The overall domains within the postulated pathway were (i) patient and environmental factors to (ii) comorbidities to (iii) left ventricular ejection fraction to (iv) symptoms to (v) functional limitations to (vi) patient-rated health. We tested the direct relationships between the individual domains and patient-rated health and the indirect relationships that were explained by intermediary domains. We did not include the final domain in Wilson and Cleary’s model (overall quality of life), as this includes non-health-related factors.

### Patient and environmental factors

HF patient factors were age, sex, marital status (single or married/partner), body mass index (BMI), smoking status (current or former/none), HF duration (≥6 or <6 months), heart rate, haemoglobin, devices, and prescription of HF optimising drugs (beta blockers, angiotensin converting enzyme inhibitors [ACEis], angiotensin II receptor blockers [ARBs]) and diuretics. Environmental factors were healthcare provider (inpatient or outpatient) and speciality (cardiology or medicine). Of the continuous variables, age and heart rate values were all within the plausible range, and BMI and haemoglobin were truncated at the boundaries of their respective plausible ranges (<10 values truncated in total).

### Bio-physiological status

HF biological status was based on left ventricular ejection fraction defined as reduced (<40%), midrange (40%–49%), or preserved (≥50%). For the sequences of regressions analyses, ejection fraction was dichotomised as ≥40% or <40%. Cardiovascular comorbidities were hypertension, dilated cardiomyopathy (DCM), valve disease, atrial fibrillation (AF), and ischemic heart disease (IHD), and non-cardiovascular comorbidities were diabetes, chronic obstructive pulmonary disease (COPD), and chronic kidney disease (CKD). CKD was defined by an estimated glomerular filtration rate of <60 ml/min/1.73 m^2^.

### Symptoms and functional limitations

Previous evidence has shown only weak associations between HF biological status and shortness of breath (SOB) [18–20], but the influence of other conditions or symptoms on this relationship has not been considered. Two of the most frequently reported HF symptoms are SOB and fatigue [21]. However, HF patients also commonly experience pain and low mood [8,21,22], and these symptoms negatively impact functional performance [23]. So, for the symptoms domain, we included pain and anxiety or depression dimensions from the EQ-5D questionnaire, together with patient-reported SOB and fatigue. For functional limitations, the ‘usual activities’ EQ-5D dimension was selected, which is most inclusive of a range of physical, emotional, and social functions. SOB and fatigue were dichotomised as ‘no or slight’ and ‘marked or severe’, and pain, anxiety or depression, and functional limitations were dichotomised as ‘no problems’ or ‘any problems’.

### Patient-rated health

Patient-rated health is a known predictor of mortality in HF [24]. In this study, patient-rated health was based on the EQ-VAS, which is a numerical scale from 0 (worst health imaginable) to 100 (best health imaginable) [25]. The EQ-5D is valid, comparable to other general [26] and specific quality of life tools [27], and widely used in HF clinical trials [9].

### Statistical analysis

#### Descriptive analysis

We dichotomised patient-rated health (EQ-VAS) at the population mean (worse health and better health) to describe patient and clinical characteristics in a contingency table. Summary statistics are means and standard deviations for continuous data with symmetrical distribution, whilst skewed continuous data are described by medians with interquartile ranges. Dichotomous data are summarised by frequencies and percent prevalence.

#### Sequences-of-regressions analysis

We used sequences of regressions [28], a subclass of graphical Markov models, to estimate the associations between HF comorbidities and patient-rated health, separating direct from indirect associations via symptom burden and functional limitations. This is a multivariate statistical technique that extends path analysis and provides a novel strategy for describing complex interrelations by jointly modelling multiple outcomes and background factors (S1 Methods). The model was built by fitting ordered sequences of logistic or linear regression models for each variable in the different domains organised a priori into the HF health model (Fig 2). Each variable in turn, starting with patient-rated health and working backwards, was fitted into a regression model with all the variables to its left-hand side. The regression model that best described the data was selected by comparing nested models with different combinations of explanatory variables. The aim of the model was to partition the total effects of cardiovascular and non-cardiovascular comorbidities, left ventricular ejection fraction, symptoms, and functional limitations on patient-rated health into direct effects (not explained by any of the intermediary factors) and indirect effects (partially explained by intermediary factors such as symptoms and functional limitations). These pathways of association are represented in regression graphs that display all the variables, with two variables linked by an arrow if they are associated. An arrow emerging from a selected explanatory variable and pointing to a response variable represents a direct association. A sequence of connected arrows between 2 variables represents an indirect association. Partial regression coefficients were used to quantify the relative importance of the associations depicted by the arrows in the graph. All response variables except EQ-VAS are binary, thus odds ratios (ORs) with 95% confidence intervals for these are reported. For EQ-VAS as the response variable, the partial regression coefficients represent the mean differences in score for a 1-unit increase in the explanatory variable, and these are reported with 95% CIs.

To check for non-linear terms in the regression models, quadratic or interaction terms were included whenever these were highlighted by screening plots appropriate for checking the assumptions of the multivariate normal distribution. The level of statistical significance was set at *P* < 0.01, and analyses were performed using StataMP 13. There were very small proportions of missing values for a few explanatory and intermediate variables (Table 1); these are unlikely to bias the estimation of the model, which used all the data available when estimating single regression models, thus assuming a missing at random mechanism.

**Table 1:** Characteristics by patient-rated health.

Characteristic	Total (*N* = 10,575)	Worse patient-rated health* (*N* = 5,430)	Better patient-rated health* (*N* = 5,145)	Missing (%)
**Patient and environmental factors**				
Age, years	74 [65–81]	75 [67–81]	72 [64–80]	—
Women	3,473 (32.8)	1,957 (36.0)	1,516 (29.5)	—
Single	3,958 (38.6)	2,205 (41.7)	1,753 (35.3)	3.1
Body mass index	26.7 [24–30]	26.7 [24–30]	26.7 [24–30]	4.6
Current smoker	1,202 (12.6)	655 (13.5)	547 (11.7)	10.0
Haemoglobin, g/l	134 ± 17	133 ± 17	136 ± 16	0.03
Ejection fraction				8.5
<40%	6,031 (62.3)	3,078 (62.2)	2,953 (62.5)	
40%–49%	2,126 (22.0)	1,034 (20.9)	1,092 (23.1)	
≥50%	1,517 (15.7)	835 (16.9)	682 (14.4)	
Heart failure ≥ 6 months	5,161 (49.1)	2,873 (53.2)	2,288 (44.7)	0.6
Heart rate	72.4 ± 14.7	73.3 ± 14.8	71.5 ± 14.6	0.01
Beta blocker	9,330 (88.5)	4,788 (88.6)	4,452 (88.5)	0.4
ACEi or ARB	9,520 (90.4)	4,812 (89.1)	4,708 (91.7)	0.4
Diuretic	8,138 (77.4)	4,446 (82.4)	3,692 (72.1)	0.6
Device	1,440 (13.8)	772 (14.4)	668 (13.1)	1.2
Cardiology care	5,506 (59.5)	2,731 (57.3)	2,775 (61.7)	12.5
Inpatient	2,746 (26.0)	1,466 (27.0)	1,280 (24.9)	—
**Comorbidities**				
Ischemic heart disease	5,418 (53.9)	2,696 (52.4)	2,181 (44.5)	5.0
Atrial fibrillation	5,064 (48.1)	2,739 (50.7)	3,325 (45.4)	0.5
Hypertension	5,311 (51.5)	2,745 (52.1)	2,566 (51.0)	2.5
Dilated cardiomyopathy	1,599 (15.7)	750 (14.4)	849 (17.1)	3.5
Valve disease	603 (5.8)	322 (6.0)	281 (5.5)	1.3
Diabetes	2,518 (23.9)	1,447 (26.8)	1,071 (20.9)	0.5
Chronic obstructive pulmonary disease	1,813 (17.4)	1,101 (20.7)	712 (14.0)	1.7
Chronic kidney disease**	4,646 (43.9)	2,619 (48.2)	2,027 (39.4)	—
**Symptoms**				
Shortness of breath				7.4
Any	8,354 (85.3)	4,623 (92.7)	3,731 (77.7)	
Marked or severe	3,475 (35.0)	2,528 (50.7)	947 (19.7)	
Fatigue				7.5
Any	8,281 (84.6)	4,648 (93.1)	3,633 (75.8)	
Marked or severe	3,400 (34.7)	2,494 (49.9)	906 (18.9)	
Pain (any)	5,065 (51.9)	3,206 (64.4)	1,859 (38.9)	7.8
Anxiety or depression (any)	4,336 (44.6)	2,932 (59.2)	1,404 (29.4)	8.0
**Functional limitations (usual activities)**	3,636 (37.2)	2,619 (52.6)	1,017 (21.3)	7.7
**Patient-rated health**	62.6 ± 19.9	47.2 ± 14.0	78.8 ± 9.7	—

Data are expressed as frequency (%), mean ± standard deviation, or median [interquartile range] (for variables with skewed distribution).

*Patient-rated health was determined by the EuroQol visual analogue scale. Patients rated their health from 0 (worst imaginable health state) to 100 (best imaginable health state). We dichotomised this at the mean value (<63, ≥63) to describe ‘worse’ and ‘better’ patient-rated health for our sample.

**Defined by estimated glomerular filtration rate < 60 ml/min/1.73 m^2^.

ACEi, angiotensin converting enzyme inhibitor; ARB, angiotensin II receptor blocker.

## Results

### Study population characteristics

There were 10,575 HF patients with EQ-VAS recorded at inclusion in the register, and patient characteristics are presented in Table 1 (comparison to patients in the overall register including those without EQ-VAS recorded can be seen in S1 Table). Median age was 74 years (IQR 65–81), and 33% were women. A quarter (26%) of patients were inpatients, and most (90%) were prescribed HF optimising drug therapies. Prevalence of symptoms and functional limitations was high, with 85% patients experiencing SOB and fatigue and over a third (35%) reporting marked or severe intensity of both symptoms. Approximately half reported pain (52%) and anxiety (45%). Functional limitations were reported by 37% of HF patients, and the mean EQ-VAS score was 63 (SD 20).

### Cardiovascular and non-cardiovascular comorbidities

**Fig 3:**
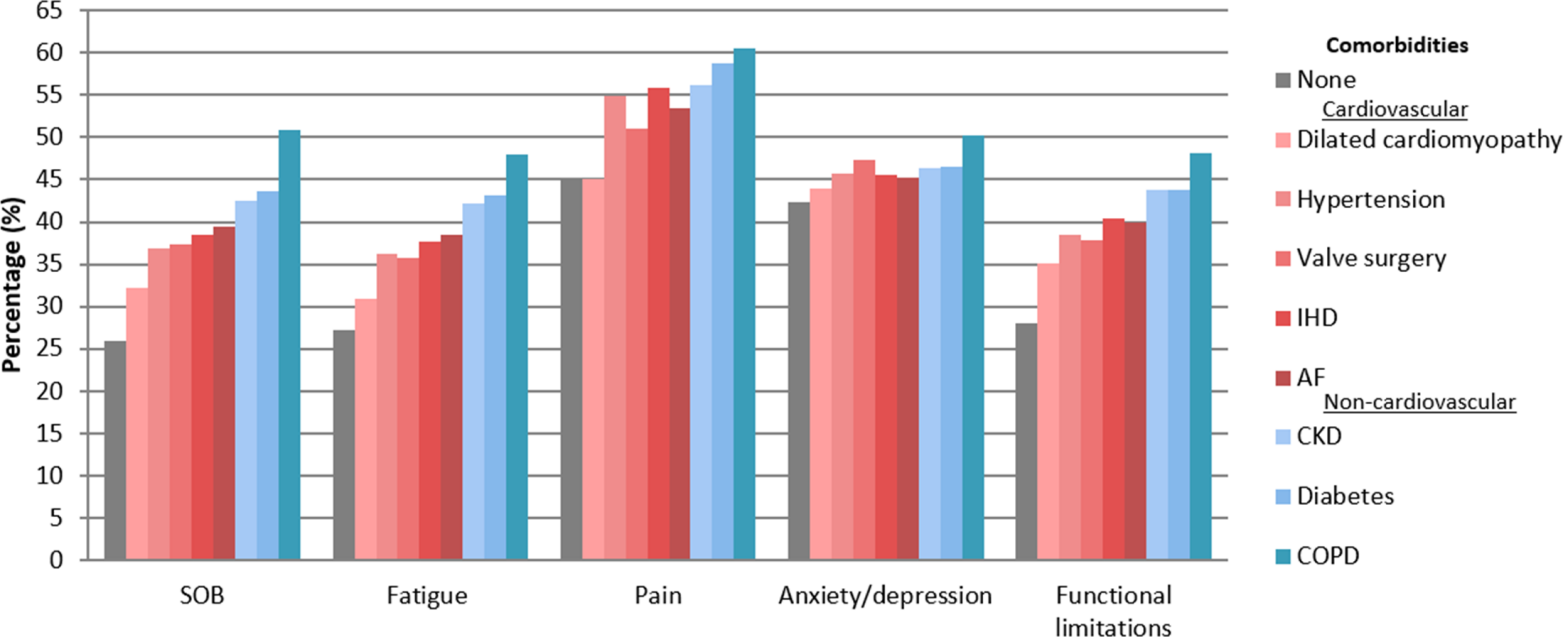
Prevalence of symptoms and functional limitations in heart failure by comorbidity. Shortness of breath (SOB) and fatigue show patients with marked or severe symptoms. Pain, anxiety or depression, and functional limitations show patients with ‘any’. AF, atrial fibrillation; CKD, chronic kidney disease; COPD, chronic obstructive pulmonary disease; IHD, ischemic heart disease.

The prevalence of cardiovascular comorbidities was high, with half of HF patients having AF (48%), hypertension (52%), and IHD (54%). Non-cardiovascular comorbidities were also frequent: 44% of patients had CKD, 17% had COPD, and 24% had diabetes. There were 415 (3.9%) HF patients without any comorbidities. These patients were on average 10 years younger, mostly outpatients (83%), and much more likely to have shorter duration of HF (<6 months) than HF patients with comorbidities (82% versus 50%). The prevalence of symptoms and functional limitations was higher for HF patients with comorbidities than for those without and differed between those with cardiovascular and non-cardiovascular comorbidities, as shown in Fig 3.

**Fig 4:**
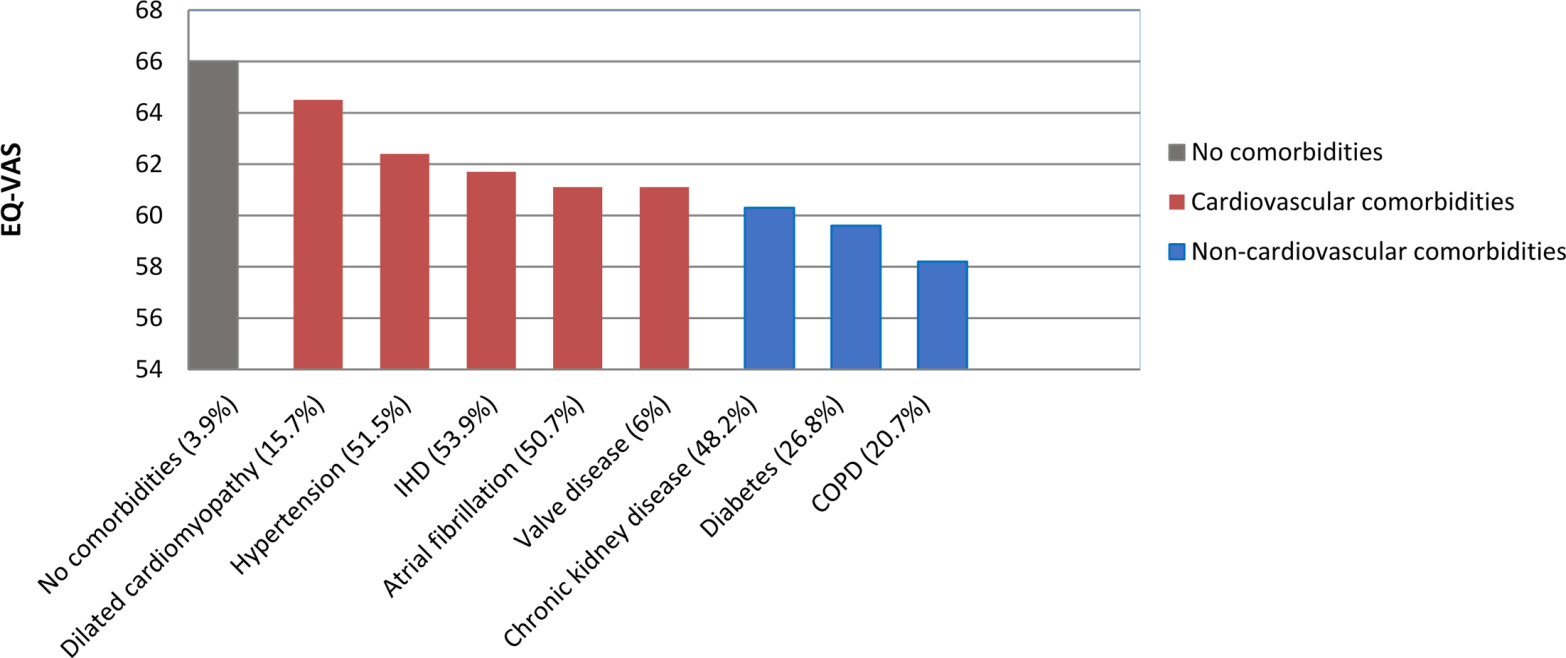
Patient-rated health (EQ-VAS) in heart failure by comorbidity. EQ-VAS was based on patient-rated health ranging from 0 (worse imaginable health state), 100 (best imaginable health state). COPD, chronic obstructive pulmonary disease; EQ-VAS, EuroQol–5 dimension visual analogue scale; IHD, ischaemic heart disease.

There was a marked increase in the prevalence of symptoms and functional limitations from HF patients with no comorbidities to those with cardiovascular comorbidities to those with non-cardiovascular comorbidities. Respectively, mean prevalence among the 3 comorbidity categories was 26%, 37%, and 46% for marked or severe SOB; 27%, 36%, and 44% for marked or severe fatigue; 45%, 52%, and 59% for pain; 42%, 46%, and 48% for anxiety or depression; and 28%, 38%, and 45% for functional limitations. Patient-rated health showed a similar pattern (Fig 4), where mean EQ-VAS score progressively declined from no comorbidities (mean EQ-VAS score, 66) to cardiovascular comorbidities (mean EQ-VAS score, 62) to non-cardiovascular comorbidities (mean EQ-VAS score, 59).

### Direct associations between all variables and patient-rated health

Summaries of the sequences of regressions fitted for the HF health model are presented in Tables 2 and S2. Patient-rated health (EQ-VAS score, 1 to 100) was directly explained by a combination of patient and environmental factors, symptoms, and functional limitations. These factors collectively explained 32% of the variance in patient-rated health (coefficient of multiple determination *R*^2^ = 0.32, 95% CI 0.31 to 0.34). There were direct negative associations with patient-rated health for older age, single status, longer HF duration, higher heart rate, prescribed diuretic, being an inpatient, having diabetes, and having symptoms and functional limitations. Care under cardiology compared to general medicine was associated with better patient-rated health (mean difference in EQ-VAS score, 1.28, 95% CI 0.54 to 2.03). Two of the strongest direct associations with worse patient-rated health were anxiety or depression (mean difference in EQ-VAS score, −18.68, 95% CI −23.2 to 14.1) and functional limitations (mean difference in EQ-VAS score, −7.51, 95% CI −8.37 to −6.65). Whilst most comorbidities had significant crude associations with patient-rated health (S3 Table), they did not have direct associations with patient-rated health once other variables including symptoms and functional limitations were considered, except for diabetes.

**Table 2:** Selected regression models describing direct and indirect associations between health factors.

Explanatory variable	Dependent variable (outcome for each model)					
	SOB, OR (95% CI)	Fatigue, OR (95% CI)	Pain, OR (95% CI)	Anxiety or depression, OR (95% CI)	Functional limitations, OR (95% CI)	Patient-rated health, relative mean difference (95% CI)
Age (per year)	1.01 (1.01, 1.02)	1.02 (1.01, 1.03)	1.01 (1.01, 1.02)	0.99 (0.98, 0.99)	1.02 (1.01, 1.03)	−0.08 (−0.13, −0.04)
Women		3.48 (1.72, 7.03)	1.39 (1.25, 1.54)	1.61 (1.46, 1.78)	1.17 (1.06, 1.30)	
Single						−1.58 (−2.33, −0.83)
Body mass index	1.04 (1.03, 1.05)	1.02 (1.02, 1.03)	1.04 (1.03, 1.05)			
Current smoker	1.19 (1.01, 1.39)	1.26 (1.09, 1.47)	1.40 (1.21, 1.62)	1.21 (1.05, 1.39)		
Haemoglobin	0.93 (0.90, 0.96)	0.94 (0.91, 0.97)	0.90 (0.88, 0.93)	0.95 (0.93, 0.98)		
Heart failure ≥ 6 months	1.39 (1.25, 1.54)	1.23 (1.11, 1.36)			1.18 (1.07, 1.30)	−1.33 (−2.08, −0.58)
Heart rate	1.01 (1.01, 1.01)	1.01 (1.00, 1.01)				−0.05 (−0.08, −0.03)
Beta blocker						
ACEi or ARB		0.70 (0.59, 0.82)				
Diuretic	1.94 (1.67, 2.24)	1.61 (1.41, 1.85)	1.29 (1.15, 1.44)	1.40 (1.25, 1.57)	1.34 (1.18, 1.52)	−1.42 (−2.35, −0.49)
Device		1.26 (1.09, 1.45)				
Cardiology	0.82 (0.74, 0.92)					1.28 (0.54, 2.03)
Inpatient	1.35 (1.20, 1.51)	1.44 (1.29, 1.60)		1.24 (1.11, 1.37)	1.28 (1.15, 1.44)	−2.04 (−2.87, −1.21)
**Comorbidities**						
Ischemic heart disease			1.25 (1.14, 1.38)	1.20 (1.09, 1.32)		
Atrial fibrillation				1.16 (1.06, 1.27)		
Hypertension						
Dilated cardiomyopathy						
Valve surgery						
Diabetes	1.17 (1.03, 1.32)	1.27 (1.13, 1.42)				−1.33 (−2.20, −0.47)
COPD	1.84 (1.62, 2.10)	1.69 (1.50, 1.91)	1.34 (1.19, 1.52)	1.21 (1.08, 1.36)	1.22 (1.08, 1.38)	
Chronic kidney disease*	1.23 (1.10, 1.38)	1.24 (1.12, 1.38)				
**Ejection fraction <40%**						
Symptoms**						
Shortness of breath					1.98 (1.74, 2.26)	−6.24 (−7.30, −5.18)
Fatigue					2.45 (2.16, 2.79)	−5.53 (−6.61, −4.45)
Pain					2.33 (2.10, 2.58)	−4.51 (−5.31, 3.71)
Anxiety or depression					10.03 (5.16, 19.50)	−18.68 (−23.22, −14.14)
**Functional limitations (problems with usual activities)**						−7.51 (−8.37, −6.65)
**Non-linear effects and interactions**						
Age (per year) and female		0.99 (0.98, 0.99)				
Age (per year) and anxiety or depression					0.98 (0.97, 0.99)	0.15 (0.08, 0.21)

The models were built by fitting ordered sequences of logistic or linear regression models for each variable adjusted by all the variables to their left-hand side in the HF health model (Fig 2). Where variables were in domains of multiple outcomes, e.g., symptoms, they were regressed on variables to their left that were not in the same domain. Variables in the same domain or to the right of the outcome variable are noted by grey shading as these would not be relevant for that component of the model. For each outcome, the regression model that best described the data was selected by comparing nested models with different combinations of explanatory variables. Patient-rated health was based on EuroQol visual analogue scale, where patients rate their health from 0 (worst imaginable health state) to 100 (best imaginable health state).

*Defined by estimated glomerular filtration rate < 60 ml/min/1.73 m^2^.

**Pain, anxiety, and functional limitations were ‘any’ compared to none, and shortness of breath and fatigue were ‘marked or severe’ compared to ‘none or slight’.

ACEi, angiotensin converting enzyme inhibitor; ARB, angiotensin II receptor blocker; COPD; chronic obstructive pulmonary disease.

### Indirect associations between HF comorbidities and patient-rated health

**Fig 5:**
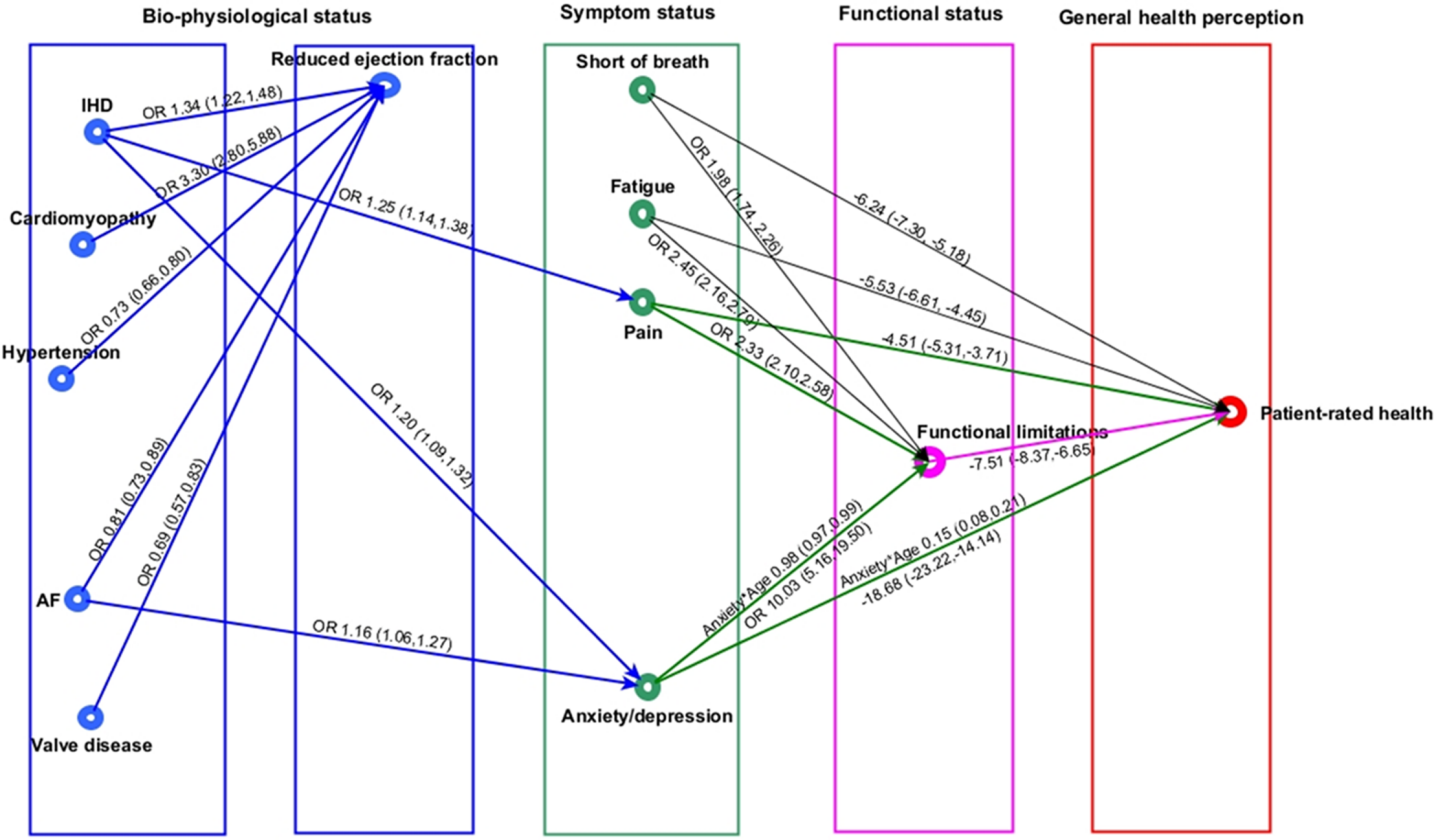
Cardiovascular comorbidities in heart failure and patient health pathway. In the regression graph an arrow is present between a response and an explanatory variable if there is a significant association (*P* < 0.01), controlling for all remaining regressors. The strength of this association is shown as OR (95% CI), if the response variable is binary, and mean difference (95% CI) in the response variable for a 1-unit increase in the explanatory variable, if the response variable is continuous. Significant interactions and non-linear relationships are also indicated. Reduced ejection fraction defined as <40%. Pain and anxiety or depression defined as ‘any problems’. Shortness of breath and fatigue defined as ‘marked or severe’, and functional limitation as ‘any’ limitation in usual activities. Patient-rated health was measured by EuroQol visual analogue scale, ranging from 0 (worst health imaginable) to 100 (best health imaginable). AF, atrial fibrillation; IHD, ischemic heart disease; OR, odds ratio.

**Fig 6:**
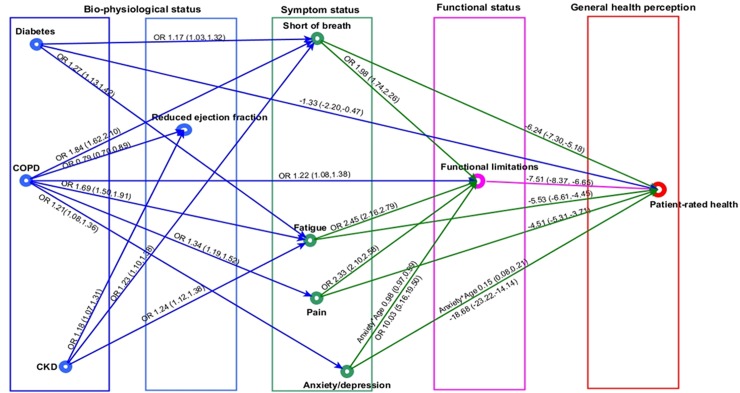
Non-cardiovascular comorbidities in heart failure and patient health pathway. In the regression graph an arrow is present between a response and an explanatory variable if there is a significant association (*P* < 0.01), controlling for all remaining regressors. The strength of this association is shown as OR (95% CI), if the response variable is binary, and mean difference (95% CI) in the response variable for a 1-unit increase in the explanatory variable, if the response variable is continuous. Significant interactions and non-linear relationships are also indicated. CKD defined as estimated glomerular filtration rate < 60 ml/min/1.73 m^2^. Reduced ejection fraction defined as <40%. Pain and anxiety or depression defined as ‘any problems’. Shortness of breath and fatigue defined as ‘marked or severe’, and functional limitation as ‘any’ limitation in usual activities. Patient-rated health was measured by EuroQol visual analogue scale, ranging from 0 (worst health imaginable) to 100 (best health imaginable). CKD, chronic kidney disease; COPD, chronic obstructive pulmonary disease; OR, odds ratio.

For simplicity of presentation, the indirect pathways between comorbidities and patient-rated health are displayed in 2 graphs (cardiovascular comorbidities in Fig 5 and non-cardiovascular comorbidities in Fig 6). Individual and environmental factors are not displayed.

### Cardiovascular comorbidities and patient-rated health

Whilst none of the cardiovascular comorbidities had a direct association with patient-rated health, 2 had indirect associations through pain, anxiety or depression, and functional limitations (Fig 5). There were no associations between any of the cardiovascular comorbidities and SOB or fatigue. The association between AF and patient-rated health was explained by an increased risk of anxiety or depression (OR 1.16, 95% CI 1.06 to 1.27), which in turn was associated with increased risk of functional limitation (OR 10.03, 95% CI 5.2 to 19.5) and subsequently worse patient-rated health (mean difference in EQ-VAS score, −7.51, 95% CI −8.37 to −6.65). IHD was associated with pain (OR 1.25, 95% CI 1.14 to 1.38) and anxiety or depression (OR 1.20, 95% CI 1.09 to 1.32), which were both associated with functional limitations and subsequent worse patient-rated health. There were also direct associations between pain and worse patient-rated health (mean difference in EQ-VAS score, −4.51, 95% CI −5.31 to −3.71) and anxiety or depression and worse patient-rated health (mean difference in EQ-VAS score, −18.68, 95% CI −23.22 to −14.14) that were not explained by functional limitations. DCM, hypertension, and valve disease in HF were not associated directly or indirectly with symptoms, functional limitations, or patient-rated health. Whilst all cardiovascular comorbidities were directly associated with ejection fraction, there was no associations between ejection fraction status and any of the other symptoms or health domains.

### Non-cardiovascular comorbidities and patient-rated health

Diabetes, COPD, and CKD had indirect associations with patient-rated health through symptoms and functional limitations, but there were variations in symptom associations (Fig 6). Diabetes and CKD were significantly associated with marked or severe SOB (OR 1.17, 95% CI 1.03 to 1.32, and OR 1.23, 95% CI 1.10 to 1.38, respectively) and fatigue (OR 1.27, 95% CI 1.13 to 1.42, and OR 1.24, 95% CI 1.12 to 1.38) compared to HF patients without these comorbidities. However, there were no associations for either comorbidity with pain and anxiety or depression. COPD, in contrast, was associated with all 4 symptoms: SOB (OR 1.84, 95% CI 1.62 to 2.10), fatigue (OR 1.69, 95% CI 1.50 to 1.91), pain (OR 1.34, 95% CI 1.19 to 1.52), and anxiety or depression (OR 1.21, 95% CI 1.08 to 1.36). As with pain and anxiety or depression, SOB and fatigue were associated with functional limitations (SOB, OR 1.98, 95% CI 1.74 to 2.26; fatigue, OR 2.45, 95% CI 2.16 to 2.79) (and subsequent worse patient-rated health) and also had direct associations with patient-rated health (SOB, mean difference in EQ-VAS score, −6.24, 95% CI −7.30 to −5.14; fatigue, mean difference in EQ-VAS score, −5.53, 95% CI −6.51 to −4.45) that were not explained by functional limitations. Again, ejection fraction did not explain any of the relationships between comorbidities and the other patient-rated health domains.

### Indirect pathways between patient/environmental factors in HF, comorbidities, and patient-rated health

There were indirect pathways through comorbidities to patient-rated health for age, sex, BMI, smoking, speciality, and healthcare provider. With few exceptions, older age, higher BMI, and being an inpatient rather than an outpatient were associated with higher levels of cardiovascular and non-cardiovascular comorbidities. Women were less likely to have cardiovascular comorbidities and more likely to have COPD and CKD than men. Compared to general medicine, cardiology care was associated with the presence of some but not all cardiovascular comorbidities as well as CKD (see S2 Table). ACEi or ARB prescription in HF patients was associated with reduced fatigue compared to patients not prescribed an ACEi or ARB (OR 0.70, 95% CI 0.59 to 0.82), and those with a device had increased fatigue compared to patients without a device (OR 1.26, 95% CI 1.09 to 1.45), but there were no other significant associations between HF optimising therapies (pharmacology or devices) and symptoms, functional limitations, or patient-rated health (Table 2).

## Discussion

Our study used an a priori evidence-informed health model to investigate the potential pathways linking comorbidities with patient-reported symptoms, functional limitations, and patient-rated health in a large population-based registry of over 10,000 patients. The importance of elucidating these pathways for clinical management is the potential to improve HF prognosis by tailoring interventions to an individual patient’s risk, pathology, and health. Uniquely, by using sequences of regressions to separate out direct and indirect associations, we found that the potential pathways to HF patient health are different for cardiovascular compared to non-cardiovascular comorbidities and among specific comorbid diseases. Importantly, with minor exception, HF optimising therapies were not associated with symptoms, functional limitations, or patient-rated health. These key findings provide the evidence for a step-change in understanding and testing mechanisms between HF and overall patient-rated health and for developing precision medicine that targets specific comorbidities and associated symptoms.

There are 3 key findings with important implications for clinical care. First, the main symptoms associated with cardiovascular comorbidities were pain and anxiety or depression and not SOB and fatigue. Notably, despite the high prevalence and severity of SOB and fatigue in HF generally, around half of all patients reported pain and anxiety or depression, which is similar to levels found in hospitalised HF patients [21]. Furthermore, anxiety or depression had the strongest associations, out of all 4 symptoms, with functional limitations and patient-rated health. Chronic depression was poorly reported in the SHFR, so the number of people with chronic or new depression symptoms is likely a lot higher. This means that the association between depression and functional limitation or poor health may be even greater. Yet, it has long been recognised that psychosocial factors such as anxiety and depression are infrequently assessed or treated in HF clinical practice, with only a minority of HF patients with depression prescribed antidepressant drugs or counselling [22] or referred for cognitive behavioural therapy [29]. Pain is also poorly managed in HF patients, with relatively low use of analgesics or opioids [22].

Second, the 3 common non-cardiovascular comorbidities (COPD, diabetes, and CKD) were associated with the highest overall symptom burden and with more severe symptoms than were associated with cardiovascular comorbidities or with no comorbidities. The predominant symptoms associated with COPD, diabetes, and CKD were SOB and fatigue, although COPD was associated with all 4 symptoms. Explanations for the increase in cardiovascular-related symptoms with non-cardiovascular diseases are multiple and varied. It is likely that comorbidities interact with the HF via conflicts in medications, efficacy of interventions, patient self-care, or shared risk factors such as obesity and reduced exercise [30].

The implications of both these findings are important. The symptom focus in HF guidelines and clinical practice is usually on SOB, leg swelling, and fatigue [6]. These symptoms collectively drive clinical management, which focuses on cardiovascular status and associated optimisation of therapies, particularly for HF with reduced ejection fraction [31]. Whilst the management of comorbidities is prioritised for HF patients with preserved ejection fraction, this likely reflects the failure of the numerous Phase II and III trials to show any convincing evidence of benefit from standard HF treatments in this group [32]. So, despite the emphasis on comorbidities for HF patients with preserved ejection fraction, symptoms are not specifically addressed in guidelines, and the comorbidities included are poorly aligned with symptom status.

Our findings show that a potential mismatch exists between these guidelines and patient-rated health, with increased SOB and fatigue being driven by non-cardiovascular status and pain and anxiety or depression being driven by cardiovascular status. This indicates that, for HF patients’ health to improve, new interventions for common HF symptoms need to include the most prevalent non-cardiovascular comorbidities and that management of pain and anxiety or depression needs to become part of routine guideline-driven care and would be an important addition to clinically relevant end-points in clinical trials.

Our third key finding was that although HF optimising therapies were prescribed for most SHFR patients, with minor exception, these therapies were not associated directly or indirectly with comorbidities, symptoms, functional limitations, or patient-rated health. Previous evidence has found only modest benefit from HF pharmacological treatment for quality of life in HF [33], but we also found a poor relationship between therapies and both symptoms and functional limitations. Given the strong associations between patient-rated health and outcomes in HF [3,4], this raises the question of whether HF management needs to be more precise and tailored to the individual patient’s comorbidity and related symptom status. HF patients are older (median age 74 years) and experience multiple conditions and a wide range of symptoms, as indicated by our study, which means that novel multi-condition and multi-disciplinary approaches to care will be required to improve their prognosis and health [34]. However, such structured or multi-disciplinary care is not part of current guideline recommendations [22].

### Strengths and limitations

The SHFR includes patients from most (>85%) hospitals in Sweden as well as primary care units and covers the full range of patients with preserved and reduced ejection fraction as well as those with comorbidities, who are often excluded from clinical trials. The availability of such data meant that an a priori HF health model could be hypothesized and tested to provide a detailed concept of the potential pathways between comorbidities and patient-rated health in HF. It is important to note that health measures were only available for a quarter of all patients entered into the register. Whilst the SHFR was created in 2005, EQ-5D has only been included as part of baseline registrations since February 2008. There has been a slow increase since then in the proportion of records completed, particularly in the inpatient setting. Consequently, our sample was predominantly based in the outpatient setting, and patients were slightly younger, more often men, more often married, with a reduced ejected fraction, and more frequently prescribed HF optimising medications. This could imply an underestimation of poor patient-rated health in our study, as older age groups, women, and patients in hospital reported more symptoms than their younger, male, and outpatient counterparts. The impact of such differences on the complex relationships between comorbidities and health is difficult to estimate; we took account of these factors in the model, but we cannot rule out unmeasured confounding.

Whilst patient-rated health in our study was based on EQ-VAS, which is a simple, valid, and reliable measure of patient’s own health, comorbidities and symptoms were based on clinical recording, which can be subject to misclassification, leading to under-ascertainment. That said, any such misclassification is likely to bias the associations towards the null value. We used sequences of regressions for our analyses, meaning that direct and indirect associations between variables could be exposed that are often hidden by conventional statistical analyses. However, as with any method of analysis, the validity of any model depends on having considered the most influential explanatory variables at the outset. The study was retrospective and observational, with few missing data, but an important limitation was the lack of some relevant variables that might impact health such as social status/deprivation, emotional, and spiritual measures. However, our intention was to uncover the indirect relations between comorbidities and patient-rated health through subjective patient-reported health measures as opposed to producing a definitive HRQoL model, and we did use a range of clinical, patient, environmental, and biological variables based on an a priori hypothesized model. Although the investigation used a cross-sectional design, it provides novel and original findings for a HF health model that can be further tested in prospective studies and externally validated in different countries to assess the consistency of relationships postulated by the model.

### Conclusions

In HF, distinct health pathways exist among cardiovascular and non-cardiovascular comorbidities that are not influenced by HF optimising therapies. Our HF health model reveals the potential interplay of different factors that underpin health in HF. These findings highlight the need to refocus on person-centred HF care and consider the specific mechanisms that contribute to overall patient health, and to design effective interventions that target the comorbidities and the range of symptoms with the biggest impact on health.

## Supporting information

S1 Checklist Reporting guidelines checklist based on RECORD statement. (DOCX)

S1 Methods Supplementary details on sequences of regressions. (DOCX)

S1 Proposal SHFR data request proposal. (DOCX)

S1 Table Characteristics of the patients included in the study compared to overall SHFR. (DOCX)

S2 Table Selected regression models from regression graph describing direct and indirect associations between personal, biological, and physiological factors and general patient-rated health perception. (DOCX)

S3 Table Crude associations between all variables and patient-rated health. (DOCX)

## Abbreviations

ACEi: angiotensin converting enzyme inhibitor
ARB: angiotensin II receptor blocker
AF: atrial fibrillation
BMI: body mass index
CKD: chronic kidney disease
COPD: chronic obstructive pulmonary disease
DCM: dilated cardiomyopathy
EQ-5D: EuroQol–5 dimension
EQ-VAS: EuroQol visual analogue scale
HF: heart failure
HRQoL: health-related quality of life
IHD: ischemic heart disease
OR: odds ratio
SHFR: Swedish Heart Failure Registry
SOB: shortness of breath
