# Supplementary material for: Comorbidity health pathways in heart failure patients: A sequences-of-regressions analysis using cross-sectional data from 10,575 patients in the Swedish Heart Failure Registry
Source: PLoS Med. 2018 Mar 27;15(3):e1002540. doi: 10.1371/journal.pmed.1002540 (PMC5870940; doi:10.1371/journal.pmed.1002540)
Supplement: S1 Methods — (DOCX) [file pmed.1002540.s002.docx]

# S1 Supplementary methods

**Sequences of Regressions**

Sequences of Regressions,(1-3) a subclass of graphical Markov models,(4) is a multivariate statistical technique that extends path analysis.(5) We used this approach to build a multivariate model for patient-rated health, intermediate sets of multiple response variables (including functional ability, symptoms, and biological/physiological factors) and background factors (person/environment). The primary aim of the analysis was to describe direct and indirect associations between comorbidities and patient-rated health. An initial ordering of the variables (**Figure 2 – main text**) compatible with Wilson and Clearly’s theoretical model (**Figure 1 – main text**) defines the model as follows:

The variables located on the right are responses to those located to their left, and variables collocated within boxes are symmetrically associated, thus:

1. The last block contains patient-rated health which is a response variable to all variables positioned on its left (functional limitation, symptoms, ejection fraction, comorbidities, patient/environmental factors).
2. The previous block contains functional limitation which is a response variable for all those positioned on its left (symptoms, ejection fraction, comorbidities, patient/environmental factors).
3. The symptoms block contains multiple response variables for ejection fraction, comorbidities and patient/environmental factors.
4. Ejection fraction is a response variable for comorbidities and patient/environmental factors
5. Comorbidities are multiple response variables to patient/environmental factors which are background variables.

The model was built by fitting ordered sequences of logistic or linear regression models depending on whether the outcome was binary or continuous. The regression model that best described the data was selected by comparing nested models with different combinations of explanatory variables.

Sequences of regression models are characterized by a graph with nodes representing variables, arranged in blocks of multiple response variables and background factors, and connected by lines or arrows:

1. An arrow emerging from an explanatory variable and pointing to a response variable represents a *direct* association,
2. a sequence of connected arrows between two variables an *indirect* association (i.e. an association between two variables with intermediate explanatory variables)

Partial correlation coefficients were used to quantify the relative importance of associations depicted by the arrows in the graph and were presented as odds ratios (OR) for binary outcomes and mean differences for continuous outcomes with 95% confidence intervals. The presence or absence of an arrow between two variables located in different boxes indicates whether they were associated or not after partitioning out the effects of all their combined explanatory variables. However, the absence of a line between any two variables within a box does not have the usual independence interpretation because the corresponding associations were not estimated.

Although the model fitting required multiple statistical tests, the components of the model reflect distinctive relations of interest, arising from a theoretical framework, whose interpretation is of interest on their own. Therefore, the interpretation of each significance level reported is valid, and adjustment for multiple testing is not required(6).

The graphical Markov modelling approach, which is an extension to path analysis, allows the inclusion of continuous and discrete variables, nonlinear associations and a number of background variables. Indirect and direct associations can be obtained and unlike structural equation models, graphical Markov models benefit from being able to accommodate associations that are nonlinear or interactive(7). Graphical Markov modelling is a helpful approach to assess whether the data are consistent with a postulated generating mechanism reflected by the initial ordering of the variables.(8)

(1) Wermuth N, Sadeghi K. Sequences of regressions and their independences.Test 2012;21(2):215-252.

(2) Wermuth N, Cox D. Concepts and a case study for a flexible class of graphical Markov models. In: Becker C, Fried R, Kuhn S, editors. Robustness and complex data structures: festschrift in honour of Ursula Gather. Germany: Springer 2013;331-350.

(3) Solis-Trapala I, Schoenmakers I, Goldberg GR, Prentice A, Ward KA. Sequences of regressions distinguish nonmechanical from mechanical associations between metabolic factors, body composition, and bone in healthy postmenopausal women. The Journal of Nutrition 2013;146:846-854.

(4) Cox D, Wermuth N. Multivariate Dependencies: Models, Analysis and Interpretation. Oxford: Chapman & Hall;1996.

(5) Wright S. The Method of Path Coefficients. 1934;:161-215.

(6) Cox D, Wermuth N. Multivariate dependencies, models, analysis and interpretation. Boca Raton (FL): Chapman & Hall/CRC; 1996.

(7) Hardt J, Petrak F, Filipas D, Egle UT. Adaptation to life after surgical removal of the bladder-an application of graphical Markov models for analysing longitudinal data. Stat Med 2004;23(4):649-666.

(8) Hemingway H. States, rates and traits: prognosis research and chronic stable angina. Heart 2009;95:439-440.
