# Supplementary material for: Comorbidity health pathways in heart failure patients: A sequences-of-regressions analysis using cross-sectional data from 10,575 patients in the Swedish Heart Failure Registry
Source: PLoS Med. 2018 Mar 27;15(3):e1002540. doi: 10.1371/journal.pmed.1002540 (PMC5870940; doi:10.1371/journal.pmed.1002540)
Supplement: S1 Proposal — (DOCX) [file pmed.1002540.s003.docx]

# S1 SHFR data-request proposal

**Title: Investigating associations between HF comorbidity and self-reported symptoms and quality of life: Swedish Heart Failure study**

Proposer: *Claire Rushton NIHR Doctoral Fellow & cardiology nurse*

Team: *Professors Anna Strömberg, Tiny Jaarsma, Ulf Dahlström and Umesh T. Kadam and PhD student Åsa Johansson*

**Brief background**

Current published outputs on Heart failure (HF) comorbidity from the Swedish Heart Failure study identified include a conference abstract and 3 relevant papers.

1. Norhammar A et al. Impact of diabetes mellitus on long-term prognosis in patients with ischemic heart failure: a report from the Swedish Heart Failure Registry (S-HFR) (conference presentation).

2. Cermakova P et al. Heart failure and dementia: survival in relation to types of heart failure and different dementia disorders. Eur J Heart Fail. 2015 Jan 10. doi: 10.1002/ejhf.222.

3. Johansson I et al. Is the prognosis in patients with diabetes and heart failure a matter of unsatisfactory management? An observational study from the Swedish Heart Failure Registry. Eur J Heart Fail. 2014 Apr;16(4):409-18.

4. Holmström A et al. Increased comorbidities in heart failure patients ≥ 85 years but declined from >90 years: data from the Swedish Heart Failure Registry. Int J Cardiol. 2013 Sep 10;167(6):2747-52.

Heart failure is associated with poor quality of life which is itself associated with hospital admissions and death. HF patients commonly suffer from additional comorbid diseases which are known to increase the risk of hospital admission and death, but little is known about how comorbidities influence quality of life outcomes. This evidence gap needs to be investigated so that new interventions can be developed in the future to improve quality of life and prevent deterioration in this high risk HF comorbidity group.

**Aim & Objectives**:

The overall aim is to investigate the influence of comorbidities on symptoms, functional capacity quality of life outcomes in heart failure. The objectives using the Swedish HF registry data will be to investigate:

i) the association between cardiovascular disease (CVD) multimorbidity and non-CVD comorbidities and patient reported symptoms, physical function and quality of life using baseline data

ii) whether the association between cardiovascular disease multimorbidity and non-CVD comorbidities and patient reported outcomes is modified by heart failure management.

**Method:** Observational cohort design using the Swedish Heart Failure Registry (S-HFR).

The study proposes to investigate HF patient reported symptoms, physical function and quality of life measures at baseline (entry onto the register). Two HF groups will be defined:

1. CVD cardiovascular multimorbidity definition will include HF patients with prior history of hypertension, AF/flutter, MI, heart valve disease and dilated cardiomyopathy.
2. Non-CVD comorbidity will include depression, diabetes, COPD, chronic kidney disease, cancer, dementia and other undefined comorbidity.

**Database and Analysis:** The S-HFR database contains information on 50,000 patients with data on HF, comorbidity and patient-reported outcome measures (PROMs). Patient factors will be investigated for their mediator and moderator effects on the association between the comorbidities and PROMs using linear and logistic regression methods.

Linear regression will be used to measure the strength of association between the two HF groups and EQ-5D score, adjusting for patient and HF clinical characteristics. Ordinal logistic regression will be used to measure the strength of association between the two HF groups and other symptom and physical function outcomes, adjusting for patient and HF clinical characteristics. Comorbid effects on one year patient reported outcomes will be stratified by heart failure drugs and procedure management.

*Exposures*: Cardiovascular (MI, Hypertension, AF/flutter, Heart valve disease, Dilated cardiomyopathy) and non-cardiovascular comorbidities (depression, diabetes, COPD, chronic kidney disease, cancer, dementia).

*Patient factors and Modifiers*: Procedures (revascularisation, devices, heart valve), drugs (ACEi/ARB, beta blocker). Symptoms (SOB, fatigue, pain/discomfort, anxiety/depression), physical function (mobility, self-care, usual activities)

*Outcomes*: quality of life (EQ5D scale).

**Clinical key questions this project will address are:**

- Which HF comorbidity groups are important determinants of poor patient-reported outcomes and how can such high-risk groups be identified?
- Which factors in comorbidity and HF are important in influencing poor patient-reported outcomes and how might these factors be targeted in care of the patients?
- How will identifying important comorbidity and patient factors in poor HF outcomes be incorporated in heart failure education, guidelines and practical tools for patients?

**Dissemination Plan:** International workshop of nurse specialists to integrate findings into guidelines and practical tools for patient care. Dissemination through Heart Failure Nurse Curriculum, publication and conference presentations

**Expected Value to Patients:** Better trained HF nurses and patient-centred interventions aimed at improving the QoL of HF patients with comorbidity.
