# Supplementary material for: Comorbidity health pathways in heart failure patients: A sequences-of-regressions analysis using cross-sectional data from 10,575 patients in the Swedish Heart Failure Registry
Source: PLoS Med. 2018 Mar 27;15(3):e1002540. doi: 10.1371/journal.pmed.1002540 (PMC5870940; doi:10.1371/journal.pmed.1002540)
Supplement: S1 Table — (DOCX) [file pmed.1002540.s004.docx]

| **S1 Table****Characteristics of the patients included in the study compared to overall SHFR registry** | | |
| --- | --- | --- |
|  | **Overall registry** | **Patients with baseline EQ-VAS** |
| Characteristics | Total (N = 41,539) | Total (N =10,575) |
| Age, years | 77[68 to 84] | 74[65 to 81] |
| Women | 16,320(39.3) | 3,473(32.8) |
| Single | 18,481(46.3) | 3,958(38.6) |
| BMI | 26.3[23 to 23] | 26.7[24 to 30] |
| Current smoker | 4,108(12.9) | 1,202(12.6) |
| Hemoglobin | 13.1±1.8 | 13.4±1.7 |
| EF <40% | 19,673(55.4) | 6,031(62.3) |
| HF < 6months | 20,116(49.0) | 5,351(50.9) |
| Heart rate | 74.4±15.8 | 72.4±14.7 |
| Beta blocker | 35,188(85.2) | 9,330(88.5) |
| ACEi or ARB | 33,712(81.7) | 9,520(90.4) |
| Diuretic | 32,815(79.5) | 8,138(77.4) |
| Device | 5,685(13.9) | 1,440(13.8) |
| Cardiology | 20,263(53.3) | 5,506(59.5) |
| Inpatient | 23,057(55.5) | 2,746(26.0) |
| Comorbidities | | |
| IHD | 18,345(46.3) | 5,418(53.9) |
| AF | 21,009(50.9) | 5,064(48.1) |
| Hypertension | 21,560(53.1) | 5,311(51.5) |
| DCM | 4,334(10.9) | 1,599(15.7) |
| Valve disease | 2,396(5.9) | 603(5.8) |
| Diabetes | 10,248(24.8) | 2,518(23.9) |
| COPD | 7,662(18.8) | 1,813(17.4) |
| CKD | 20,832(50.2) | 4,646(43.9) |
| Data are expressed as No. (%), mean ± standard deviation or median [interquartile range] for variables with skewed distribution. BMI, body mass index ; EF, ejection fraction; HF, heart failure; ACEi or ARB, angiotensin converting enzyme inhibitor or Angiotensin II receptor blocker; IHD, ischemic heart disease; AF, atrial fibrillation; DCM, dilated cardiomyopathy; COPD, chronic obstructive pulmonary disease; CKD, chronic kidney disease (defined by estimated glomerular filtration rate <60 mls/min/m2) | | |
