# Supplementary material for: Comorbidity health pathways in heart failure patients: A sequences-of-regressions analysis using cross-sectional data from 10,575 patients in the Swedish Heart Failure Registry
Source: PLoS Med. 2018 Mar 27;15(3):e1002540. doi: 10.1371/journal.pmed.1002540 (PMC5870940; doi:10.1371/journal.pmed.1002540)
Supplement: S2 Table — (DOCX) [file pmed.1002540.s005.docx]

| S2 Table Selected regression models from regression graph describing direct and indirect associations, cont’d from table 2 (main text). | | | | | | | | | | | | | | |
| --- | --- | --- | --- | --- | --- | --- | --- | --- | --- | --- | --- | --- | --- | --- |
| Explanatory variables Dependent variables (outcome for each model) | | | | | | | | | | | | | |  |
|  | **IHD** | **AF** | | **Hypertension** | | **Cardiomyopathy** | | **Valve disease** | | **Diabetes** | | **COPD** | **CKD** | **Ejection fraction <40%** |
|  | **OR(95% CI)** | **OR(95% CI)** | | **OR (95% CI)** | | **OR(95% CI)** | | **OR(95% CI)** | | **OR(95% CI)** | | **OR (95% CI)** | **OR (95% CI)** | **OR (95% CI)** |
| Age, (per year) | 1.04(1.03,1.04) | 1.04(1.04,1.05) | | 1.04(1.03,1.04) | | 0.94(0.94,0.95) | | - | | 1.01(1.01,1.02) | | 1.03(1.02,1.03) | 1.09(1.08,1.09) | 0.98(0.97,0.98) |
| Women | 0.52(0.47,0.57) | 0.75(0.69,0.82) | | - | | 0.79(0.69,0.90) | | - | | 0.75(0.68,0.84) | | 7.61(3.62,16.03) | 1.30(1.18,1.43) | 0.62(0.56,0.69) |
| Single | 0.87(0.80,0.95) | - | | - | | - | | - | | - | | - | - | - |
| BMI | - | 1.02(1.02,1.03) | | 1.07(1.06,1.07) | | 0.98(0.67,0.99) | | 0.96(0.94,0.98) | | 1.09(1.08,1.10) | | - | 1.03(1.02,1.04) | 0.96(0.95,0.97) |
| Current smoker | - | - | | - | | - | | 0.56(0.40,0.78) | | - | | 2.73(2.36,3.15) | - | - |
| Haemoglobin | - | - | | - | | - | | - | | - | | - | - | - |
| HF < 6months | - | - | | - | | - | | - | | - | | - | - | - |
| Heart rate | - | - | | - | | - | | - | | - | | - | - | - |
| Beta blocker | - | - | | - | | - | | - | | - | | - | - | - |
| ACEi or ARB | - | - | | - | | - | | - | | - | | - | - | - |
| Diuretic | - | - | | - | | - | | - | | - | | - | - | - |
| Device | - | - | | - | | - | | - | | - | | - | - | - |
| Cardiology | - | - | | - | | 1.46(1.27,1.65) | | 1.31(1.07,1.59) | | - | | - | 1.18(1.07,1.30) | - |
| Inpatient | 1.27(1.16,1.40) | 1.19(1.09,1.31) | | - | | - | | - | | 1.50(1.35,1.66) | | 1.40(1.24,1.57) | 1.26(1.14,1.39) | 0.84(0.76,0.94) |
| Comorbidities |  |  | |  | |  | |  | |  | |  |  |  |
| IHD |  |  | |  | |  | |  | |  | |  |  | 1.34(1.22,1.48) |
| AF |  |  | |  | |  | |  | |  | |  |  | 0.81(0.73,0.89) |
| Hypertension |  |  | |  | |  | |  | |  | |  |  | 0.73(0.66,0.80) |
| DCM |  |  | |  | |  | |  | |  | |  |  | 3.30(2.80,5.88) |
| Valve disease |  |  | |  | |  | |  | |  | |  |  | 0.69(0.57,0.83) |
| Diabetes |  |  | |  | |  | |  | |  | |  |  | - |
| COPD |  |  | |  | |  | |  | |  | |  |  | 0.79(0.70,0.89) |
| CKD |  |  | |  | |  | |  | |  | |  |  | 1.18(1.07,1.31) |
| EF <40% |  |  | |  | |  | |  | |  | |  |  |  |
| Nonlinear effects and interactions | | | | | | | | | | | | |  |  |
| Age*Female |  | |  | |  | |  | |  | |  | 0.98(0.97,0.98) |  |  |
| The models were built by fitting ordered sequences of logistic or linear regression models for each variable adjusted by all the variables to their left hand side in the HF-Health model (Figure 2). Where variables were in blocks of multiple outcomes e.g. comorbidities (shown here within dotted lines) they were regressed on variables to their left that were not in the same block. Variables in the same group or to the right of the outcome variable are noted by grey shading as these would not be relevant for that component of the model. For each outcome, the regression model that best described the data was selected by comparing nested models with different combinations of explanatory variables. BMI, body mass index ; EF, ejection fraction; HF, heart failure; ACEi or ARB, angiotensin converting enzyme inhibitor or Angiotensin II receptor blocker; IHD, ischemic heart disease; AF, atrial fibrillation; DCM, dilated cardiomyopathy; COPD, chronic obstructive pulmonary disease; CKD, chronic kidney disease (defined by estimated glomerular filtration rate <60 mls/min/m2). | | | | | | | | | | | | | | |
