# Supplementary material for: Comorbidity health pathways in heart failure patients: A sequences-of-regressions analysis using cross-sectional data from 10,575 patients in the Swedish Heart Failure Registry
Source: PLoS Med. 2018 Mar 27;15(3):e1002540. doi: 10.1371/journal.pmed.1002540 (PMC5870940; doi:10.1371/journal.pmed.1002540)
Supplement: S3 Table — (DOCX) [file pmed.1002540.s006.docx]

| S3 Table Crude associations between all variables and patient health | | |
| --- | --- | --- |
|  | | |
|  | **Patient-rated health (EQ-VAS)** | ***P*** |
| Individual and environmental factors | **Mean difference (95%CI)** |  |
| Age, years | -0.14 (-0.17 to -0.11) | <0.01 |
| Women | -3.05 (-3.85 to -2.45) | <0.01 |
| Single | -2.71 (-3.49 to -1.92) | <0.01 |
| BMI | -0.06 (-0.13 to -0.01) | 0.10 |
| Current smoker | -2.39 (-3.57 to -1.20) | <0.01 |
| Haemoglobin | 1.39 (1.17 to 1.61) | <0.01 |
| EF <40% | -0.12 (-0.94 to 0.70) | 0.77 |
| HF >6months | -3.88 (-4.64 to -3.13) | <0.01 |
| Heart rate | - 0.11 (-0.14 to -0.08) | <0.01 |
| Beta blocker | 0.55 (0.65 to 1.74) | 0.37 |
| ACEi or ARB | 4.58 (3.29 to 5.86) | <0.01 |
| Diuretic | -6.34 (-7.24 to -5.44) | <0.01 |
| Device | -1.19 (-2.29 to -0.08) | 0.04 |
| Cardiology | 1.46 (0.62 to 2.29) | <0.01 |
| Inpatient | -2.61 (-1.74 to -3.47) | <0.01 |
| Comorbidities |  | |
| IHD | -1.75 (-2.52 to -0.97) | <0.01 |
| AF | -2.80 (-3.56 to -2.04) | <0.01 |
| Hypertension | -0.54 (-1.31 to -0.23) | 0.17 |
| DCM | 2.15 (1.09 to 3.21) | <0.01 |
| Valve surgery | -1.53 (-3.16 to -0.11) | 0.07 |
| Diabetes | -3.89 (-4.78 to -3.01) | <0.01 |
| COPD | -5.38 (-6.38 to -4.38) | <0.01 |
| CKD | -4.13 (-4.89 to -3.37) | <0.01 |
| Symptoms |  | |
| SOB | -16.15 (-17.30 to -15.80) | <0.01 |
| Fatigue | -16.94 (-17.69 to -16.19) | <0.01 |
| Pain | -12.42 (-13.16 to -11.67) | <0.01 |
| Anxiety/depression | -14.50 (-15.23 to -13.76) | <0.01 |
| Functional limitations | | |
| Activity limitations | -16.06 (-16.81 to -15.32) | <0.01 |
| Patient rated health was based on EQ-VAS where patients rate health from 0 (worse imaginable health state) to 100 (best imaginable health state). Pain, anxiety, and activity limitations were ‘any’ and SOB and fatigue were ‘moderate to severe’. BMI, body mass index ; EF, ejection fraction; HF, heart failure; ACEi or ARB, angiotensin converting enzyme inhibitor or Angiotensin II receptor blocker; IHD, ischemic heart disease; AF, atrial fibrillation; DCM, dilated cardiomyopathy; COPD, chronic obstructive pulmonary disease; CKD, chronic kidney disease (defined by estimated glomerular filtration rate <60 mls/min/m2); SOB, shortness of breath. | | |
